# Factors Associated with Motivation for General Medicine among Rural Medical Students: A Cross-Sectional Study

**DOI:** 10.3390/ijerph19095102

**Published:** 2022-04-22

**Authors:** Kasumi Nishikawa, Ryuichi Ohta, Chiaki Sano

**Affiliations:** 1 Faculty of Medicine, Shimane University, 89-1 Enya cho, Izumo 693-8501, Japan; k.nishikawa0324@gmail.com; 2 Community Care, Unnan City Hospital, 699-1221 96-1 Iida, Daito-cho, Unnan 699-1221, Japan; 3 Department of Community Medicine Management, Faculty of Medicine, Shimane University, 89-1 Enya cho, Izumo 693-8501, Japan; sanochi@med.shimane-u.ac.jp

**Keywords:** perception, career choice, education, primary care, family practice, general practice, Japan

## Abstract

General medicine, as the Japanese version of primary care or family medicine, is critical for healthcare in aging societies. Medical students’ perceptions of general medicine and education might be associated with changes in the number of general physicians. This study aimed to clarify the association between these perceptions and students’ preferences for general medicine. A cross-sectional survey was conducted among Japanese medical students using a questionnaire on their perceptions regarding general medicine, background, and preferences for general medicine (knowledge, interest, motivation, and intention). The response rate was 70.3% (490/697). There was a large percentage gap between interest and motivation; therefore, a logistic regression analysis was performed to investigate the cause of this difference. The perceptions that general medicine meets the needs of society and the lack of general medicine educators were positively associated with motivation to become a general physician. In contrast, perceptions of lack of exposure to general medicine beyond the curriculum and inaccessibility were negatively associated with motivation. Medical students’ motivation to pursue general medicine may increase with improvements in flexible general medicine education and accessibility. Future research should investigate the relationship between students’ perceptions of general medicine and their motivation to become general physicians through longitudinal studies.

## 1. Introduction

Physicians engaged in primary care, called general physicians in Japan, are increasingly needed in aging societies [1]. General physicians have the skills to handle a wide range of diseases, manage multiple comorbidities, and engage in community medicine [2,3]. In addition, compared to other specialists, general physicians are more rural-oriented; thus, increasing the number of general physicians may alleviate the problem of maldistribution of physicians [4]. In response to the increasing need for general physicians, a new training program was established in Japan in 2018 [5]. However, the number of physicians registered in the program has not increased significantly; for example, only 2.19% (184 of 8410) in 2018 and 2.24% (206 of 9183) in 2021 [6]. The total number of general physicians and family medicine physicians remains low in Japan [6]. Improving general medicine education and quality of practice can help in increasing the number of general physicians, which, in turn, can motivate more medical students to become general physicians.

Current medical students’ perceptions of general medicine should be understood to improve general medicine education and the quality of practice. A previous study showed that a relatively high percentage of medical students are interested in general medicine. However, the number of general physicians is low, indicating high interest and low preference for acquiring specialties in general medicine [7]. Our previous study investigated medical students’ current perceptions of general medicine to identify the gap between the number of general physicians and the motivation to become general physicians among medical students [8]. The study revealed that medical students have expectations and discouragement regarding general medicine through medical education. They were interested in general medicine in patient-centered care, a broad scope of practice, and orientation to communities. Their discouragement has been related to factors such as the lack of exposure to general medicine and an underdeveloped education [8]. However, this was a qualitative study limited to a small number of students interested in general medicine. Therefore, quantitative data on the perceptions of general medicine among medical students should be collected.

For effective general medicine education, in addition to medical students’ perceptions, their preferences for general medicine and related factors should be considered. Education in line with their preference for competency in general medicine may drive their motivation to become general physicians. In Japan, some studies have identified factors associated with a career choice in general medicine [7,9]; however, to the best of our knowledge, the association between these factors and students’ preference for general medicine has remained unclear. Medical students’ perceptions and preferences regarding general medicine may be related to their background and experiences. Previous studies have identified the background of students interested in general medicine through admission from their hometown and by a particular policy [7,9].

In Japan, there is a regional quota called “Chiikiwaku”, which aims to train doctors to work in community medicine, alleviate the shortage of doctors, and require students to work in a designated region after graduation. The availability of scholarships and the length of mandatory post-graduation years differ among universities. In 2020, 18.2% of all admissions to medical schools were Chiikiwaku [10]. In addition to Chiikiwaku, some prefectures offer scholarships similar to Chiikiwaku, for which students regularly admitted to medical schools can apply after admission [11]. Previous studies have shown that Chiikiwaku is associated with medical students’ motivation toward general medicine [8,9]. However, the association between other background factors and students’ preferences for general medicine has not been clarified. Hence, the research aimed to examine the factors regarding the medical students’ perception of general medicine associated with the motivation to become general physicians in the future. In the present situation, there are no clear association and relationship between the medical students’ perception of general medicine and their motivation for general medicine through evidence from cross-sectional and longitudinal studies. As the initial step in this study, we investigated the factors associated with medical students’ preferences for general medicine, including their perceptions of general medicine and their backgrounds. We designed and analyzed a questionnaire based on our previous study. We believe that our findings contribute to providing general medicine education that meets the preferences of medical students and enhances their interest in general medicine, providing longitudinal studies on the relationship.

## 2. Materials and Methods

### 2.1. Study Design

A cross-sectional study was conducted using a questionnaire to investigate the association between medical students’ perceptions of general medicine and their preferences for general medicine.

### 2.2. Setting

**Figure 1:**
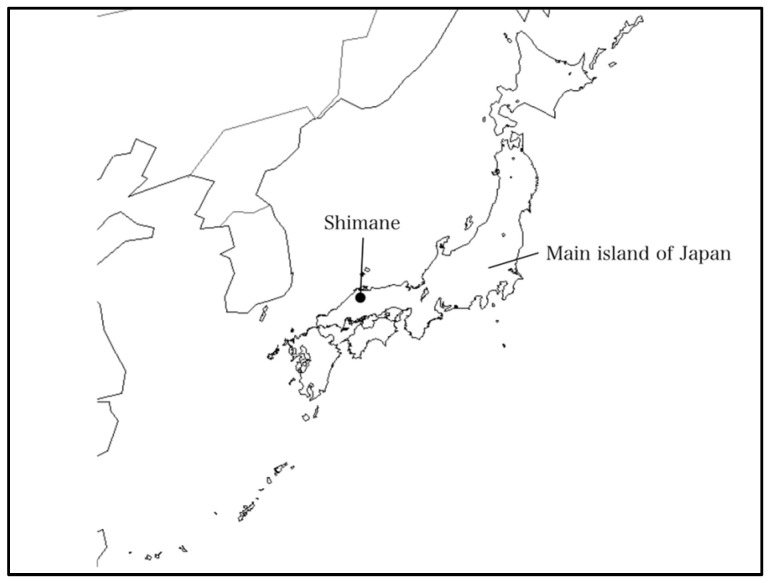
The location of the study setting.

This study was conducted among medical students at the Faculty of Medicine, Shimane University, Japan. Shimane is one of the most aged prefectures, with 34.3% of the total population of 674,000 aged 65 and over (as of 1 October 2019) [12]. Shimane is a rural area located in the southwest part of Japan (Figure 1). Shimane University is the only medical school in the region. The Chiikiwaku program for admission is available at Shimane University, in addition to its scholarships similar to Chiikiwaku. Ten hospitals, including university and community hospitals, have general medicine training programs in Shimane, and medical students can practice general medicine at those hospitals and clinics as part of the curriculum [13].

### 2.3. Participants

**Figure 2:**
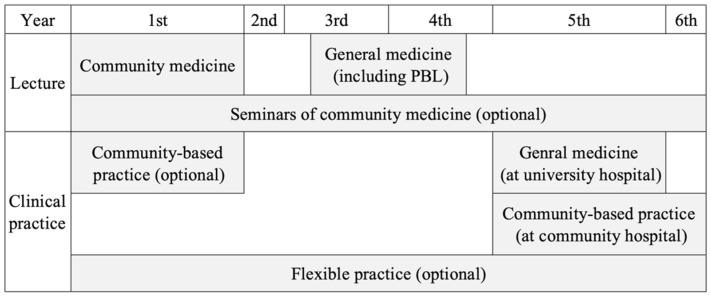
The general medicine curriculum of Shimane University Faculty of Medicine as of 2021.

In 2021, there were 697 medical students at Shimane University, comprising 108 first-grade, 120 s-grade, 110 third-grade, 99 fourth-grade, 130 fifth-grade, and 130 sixth-grade students. They learned general medicine based on the Shimane University Faculty of Medicine curriculum in 2021 (Figure 2) [8,14].

### 2.4. Data Collection

Data were collected online from 14 September to 9 November 2021, using a closed-ended questionnaire. For students in the first to fourth grades and the sixth grade, we presented the online questionnaire with a QR code at the end of a face-to-face class and asked them to complete it. Fifth-grade students were asked to complete the form through social networking sites and personal negotiations because they were in clinical practice and did not attend offline classes.

### 2.5. Dependent Variable

To measure the participants’ preference for general medicine, we used a logic model of human behavior change. According to the model, people change their behavior in four steps: knowledge, interest, motivation, and intention. The more knowledge people have about a particular behavior, the more interested and motivated they are to engage in it, leading to intention and the actual behavior itself. We asked about knowledge, interest, motivation, and intention regarding general medicine (their awareness, interest, want, and plans to practice) using the following questions: (a) Do you know about general medicine? (b) Are you interested in general medicine? (c) Are you motivated to become a general physician? (d) Do you intend to become a general physician? Participants completed these questions on a five-point Likert scale (agree, agree a little, neutral, disagree a little, disagree).

### 2.6. Independent Variables

To measure medical students’ perceptions, we used a questionnaire established based on our previous study [8]. The questionnaire consisted of 24 questions regarding medical students’ perceptions of general medicine, which were clarified through thematic analysis. These questions are listed in Table 1. Our previous study had reported 3 themes and 14 concepts, which showed that medical students had either positive or negative perceptions of general medicine and its education and the factors causing the gap between them. Thus, the questionnaire in this study consisted of three sections based on the themes of the previous study, and the 14 concepts that included overlapping meanings were divided into 24 questions. These questions were also answered on a five-point Likert scale (agree, agree a little, neutral, disagree a little, disagree).

### 2.7. Covariates

We collected the background data of the participants based on previous studies: sex (male or female), grade (grades 1 to 6), hometown (home prefecture), and Chiikiwaku (yes or no) [7,9]. Chiikiwaku included students who received prefectural scholarships and students who were admitted under the Chiikiwaku program.

### 2.8. Analysis

Fisher’s exact test was used to compare the proportions of categorical variables between groups. Variables using a five-point Likert scale were divided into two groups: (agree or agree a little = 1, neutral or disagree a little or disagree = 0). The grades were dichotomized as follows: pre-clinical years (grades one to four) and clinical years (grades five and six). Hometown was binomial based on whether it was local (Shimane) or not. Univariate analyses were performed between preference for general medicine (knowledge, interest, motivation, and intention) and other variables. In addition, we illustrate the proportion of each grade in the four preferences in graphs. The results showed a large gap between the proportions of interest and motivation in all the grades. Therefore, a multivariate logistic regression analysis of motivation was performed to identify the factors contributing to this gap. We used all 24 questions regarding perceptions of general medicine as variables, with statistically significant variables in the univariate analysis including “Hometown (Shimane or other)” and “Chiikiwaku”. Statistical significance was defined as a *p*-value < 0.05. Questions with missing data were excluded from the analysis. All statistical analyses were performed using the Easy R software (Saitama Medical Center, Jichi Medical University, Saitama, Japan) [15].

### 2.9. Ethical Considerations

Anonymity and confidentiality of the participants’ information were ensured throughout the study. All participants provided informed consent before answering the questionnaire. The research information was posted on the hospital website without any participant information. Likewise, the contact information of the hospital representative was listed on the website so that questions about the research could be answered at any time. All procedures in this study were performed in compliance with the Declaration of Helsinki and its subsequent amendments. The Unnan City Hospital Clinical Ethics Committee approved the study protocol (approval ID: 20210018).

## 3. Results

### 3.1. Characteristics of the Participants

The total response rate for the questionnaires was 70.3% (490 of 697), and the grade-wise rates for grades 1 to 6 was 86.1% (93 of 108), 63.3% (76 of 120), 81.8% (90 of 110), 92.9% (92 of 99), 48.5% (63 of 130), and 58.5% (76 of 130), respectively. Table 2 shows the characteristics of the participants according to their preference for general medicine. The total percentage for each of the preference factors, including knowledge, interest, motivation, and intention, were 72.7% (356 of 490), 66.3% (325 of 490), 36.9% (181 of 490), and 10.6% (52 of 490), respectively. The degree of knowledge regarding general medicine was significantly higher in the clinical grade students from Shimane and male students (*p* = 0.001, 0.029, and 0.019, respectively). All preferences (knowledge, interest, motivation, and intention) were significantly associated with Chiikiwaku (*p* = <0.001, 0.004, 0.001, and 0.007, respectively). In addition, motivation for general medicine was significantly associated with the Shimane group (*p* = 0.005). There were three missing data for the hometown question, which were excluded from the analysis.

### 3.2. Medical Students’ Perceptions of General Medicine and Their Preferences

Table 3 below summarizes the results of the univariate analysis of the association between medical students’ perceptions of general medicine and their preferences.

### 3.3. Medical Students’ Preferences for General Medicine

#### 3.3.1. Knowledge

The characteristics of clinical-grade, being from Shimane, male sex, and Chiikiwaku, were significantly associated with knowledge of general medicine. Regarding the perceptions of general medicine among the students, “Community-based medicine”, “Preventive care and public health”, “Homecare”, “Broad scope of practice”, “Balance between practice, education, and research”, “Meeting the needs of society”, “Diversity and development”, “Criticism from other specialists”, “Lack of educators”, and “Large Regional Disparity” were significantly related to knowledge of general medicine.

#### 3.3.2. Interest

Chiikiwaku was significantly associated with an interest in general medicine. Perceptions that were significantly related to interest were “Community-based medicine”, “Preventive care and public health”, “Broad scope of practice”, “Balance between practice, education, and research”, “Meeting the needs of society”, “Diversity and development”, “Difficulty”, “Lack of classes in the curriculum”, “Lack of educators”, and “Large Regional Disparity”.

#### 3.3.3. Motivation

Motivation to become general physicians was significantly associated with the factors being from Shimane and Chiikiwaku. The motivation was also significantly associated with the perceptions of “Community-based medicine”, “Preventive care and public health”, “Broad scope of practice”, “Meeting the needs of society”, “Diversity and development”, “Lack of educators”, and “Large Regional Disparity”.

#### 3.3.4. Intention

Chiikiwaku was significantly associated with the intention to become general physicians. Among perceptions, “Ambiguity in career path”, “Lack of clinical practice in the curriculum”, and “Criticism from other specialists” had significant correlations with intention.

### 3.4. Differences in Preferences for General Medicine by Academic Grade

**Figure 3:**
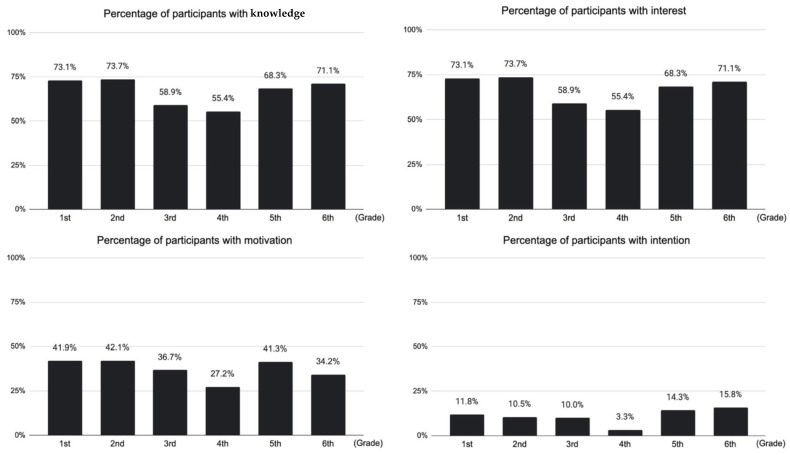
Medical students’ preferences for general medicine at each grade.

Figure 3 shows the preferences for general medicine according to the grades of the students. As for knowledge and interest, more than 50% of the students reported that they had knowledge and interest in general medicine. On the other hand, less than 50% of the students reported motivation and intention to pursue general medicine. There is, however, a considerable gap between interest and motivation. Additionally, we can see that knowledge increased in the clinical grade (fifth and sixth grades). Interest was high in the early grades (first and second), decreased in the middle grades (third and fourth), and increased again in the clinical grades (fifth and sixth).

### 3.5. Factors Associated with the Gap between Medical Students’ Interest in and Motivation for General Medicine

We performed a multivariate logistic regression analysis, focusing on motivation. Among the positive perceptions, “Meeting the needs of society” was significantly associated with medical students’ motivation to become general physicians (OR = 2.61, 95% CI: 1.02–6.68), and among the negative perceptions, “Inaccessible” was significantly associated with motivation (OR = 0.5, 95% CI: 0.28–0.88). Regarding the factors affecting students’ perceptions, “Lack of exposure beyond the curriculum” was negatively associated (OR = 0.62, 95% CI: 0.39–0.99), and “Lack of educators” was positively associated with their motivation (OR = 1.74, 95% CI: 1.10–2.76) (Table 4).

## 4. Discussion

This study found factors associated with medical students’ preferences (knowledge, interest, motivation, and intention) for general medicine, including their background and perceptions of general medicine. Our findings show a gap between the percentage of students interested in general medicine and those motivated to be general physicians. Furthermore, medical students’ perceptions of general medicine associated with this gap were identified as follows: “Meeting the needs of society”, “Lack of educators”, “Inaccessible”, and “Lack of exposure beyond the curriculum”. These findings are expected to play a crucial role in general medical education.

The background factors of the participants that were associated with knowledge of general medicine were clinical grade, hometown, sex, and Chiikiwaku. Medical students’ perceptions of general medicine associated with knowledge were community-based medicine; preventive care and public health; home care; a broad scope of practice; a balance between practice, education, and research; meeting the needs of society; diversity and development; criticism from other specialists; lack of educators; and a large regional disparity. To the best of our knowledge, this is the first cross-sectional study to examine factors associated with medical students’ knowledge of general medicine in Japan. 

The students in the clinical years were significantly more likely to know about general medicine than those in the pre-clinical years. This result suggests that exposure to general medicine at community hospitals is highly effective in understanding general medicine [16,17,18]. Knowledge was significantly associated with Chiikiwaku. This may be due to the opportunities that this system provides for students to experience general medicine, including that they are required to participate in community practice in their first and second years [17]. In addition, students with Chiikiwaku may have been more informed because they were interested in community medicine originally.

The background factor associated with participants’ interest was Chiikiwaku. Medical students’ perceptions of general medicine associated with interest were community-based medicine; preventive care and public health; a broad scope of practice; a balance between practice, education, and research; meeting the needs of society; diversity and development; difficulty; lack of classes in the curriculum; lack of educators; and a large regional disparity. More students (66.3%) were interested in general medicine than in previous studies [7,9]. Our findings reveal the percentage of medical students interested in general medicine for the first time since the department was established in 2018 [7,9]. This high percentage may reflect the growing prevalence of general medicine as a result of the establishment of the department. There was a significant association between the students’ interest in general medicine and their impression that it was challenging. Whether this difficulty leads them to be interested in it, or they are interested but find it challenging, remains unclear. In contrast, in other countries, students perceived the field as low status and lacking intellectual challenge [19,20,21]. To the best of our knowledge, this is the first study to identify a difference between foreign and Japanese medical students’ perceptions of primary care. One of the reasons for this difference is that in other countries, primary care physicians, such as general practitioners, work in limited settings, and medical students perceive them as administrators who treat only common diseases and refer more severe diseases to specialists. On the other hand, as the results of this study show, Japanese general physicians are diverse in their working style, and students’ perception of simply sending patients to the appropriate specialist department was not relevant. In addition, an association with criticism from other specialists has been shown in other countries; however, no significance was observed in this study [19,22].

Compared with the number of interested students, fewer students were motivated to be general physicians. We identified five factors associated with this gap. First, students’ perception of a lack of exposure to general medicine beyond the curriculum was negatively related to this gap. No study has clearly shown that a lack of exposure to general medicine is negatively related to medical students’ motivation to become general physicians in Japan. These data suggest that collaboration between universities and community hospitals and providing opportunities for students to be exposed to general medicine is critical to motivating them [16,17,18]. Second, inaccessibility was negatively related. This finding has not previously been reported in Japan or other countries. In addition, the results are contrary to the perception of low status in other countries, as mentioned previously [19,21]. This may be a potential reason the number of general physicians has not increased as expected. Additional research should identify the causes of this perception and design relevant interventions to motivate those who are interested but perceive inaccessibility. Third, motivation was positively related to medical students’ perception that the need for general physicians is increasing. To the best of our knowledge, there have been no studies referring to this need. A related qualitative study showed that students were motivated by observing clinical situations in which patients and their families appreciated their physicians [17]. Encouraging medical students to understand the need for general medicine may motivate them to become general physicians. Fourth, the perception of a lack of educators was positively related to motivation. Although the lack of educators was expected to be associated with students’ lower motivation to become general physicians, our findings paradoxically showed that the students were more motivated to become general physicians the more they perceived challenges in education. Previous studies have also shown a shortage of general physicians in universities [8,16]; therefore, policies to increase the number of instructors need to be promoted. Finally, we found that students who believed that general physicians had a broader scope of practice were more motivated, although this association was not significant. The competency of general physicians in handling a wide range of diseases has attracted medical students. This finding is consistent with previous studies [7,23,24]. In addition, a Japanese study showed that clinical diagnostic reasoning was significantly related to the choice of general medicine as a career [7]. Therefore, general physicians need to improve their clinical skills and educate students based on these skills to enhance their motivation.

The percentage of students who intended to become general physicians was even lower than for motivation. The background factor of the participants associated with intention was Chiikiwaku. Students’ perceptions of general medicine associated with intention were ambiguity in career path; lack of clinical practice in the curriculum; and criticism from other specialists. The intention was significantly associated with the perception that the career path of general physicians was unclear. This result suggests that ambiguity in the career path makes medical students anxious about becoming general physicians. A previous study conducted on residents also showed that anxiety about the future was a barrier to choosing a department of general medicine [25]. This may be because general medicine departments have only been established recently, and they are diverse in their workings. Therefore, students who have already decided to become general physicians need to be actively familiarized with the clinical field to let them understand how they work. 

The causal relationship between general medicine and regional quotas must be investigated. We could not determine whether Chiikiwaku as a regional quota of medical resources motivated people toward general medicine or whether those who opted for Chiikiwaku were more motivated toward general medicine in the first place. Previous studies have shown that students motivated to practice family medicine or in medically underserved areas during admission are associated with a greater willingness to practice primary care in rural areas upon graduation and after graduation [26,27]. On the other hand, another study has shown that the motivation to continue working in medically underserved areas decreases as the grade increases [27]. Therefore, longitudinal studies are needed to determine which components of regional quotas are associated with motivation toward general medicine.

The association between a lack of exposure to general medicine beyond the curriculum and motivation toward general medicine needs to be elucidated. We cannot determine whether motivated students believe there is sufficient exposure or whether the lack of exposure has reduced students’ motivation. Previous studies have revealed that community-based medical education improves students’ motivation toward general medicine [16,18,28,29,30]. In other countries, intensive educational systems provide continuous exposure to primary care from admission to graduation [30,31,32,33]; further intervention and research are needed to determine whether these systems are effective in Japan. 

The association between students’ perceptions of general medicine as inaccessible and challenging and their motivation toward general medicine needs to be investigated. We cannot determine whether inaccessibility is decreases motivation or highly motivated people are likely to have better accessibility. To the best of our knowledge, no research has been conducted on inaccessibility. Therefore, further research is needed to determine the causes of this perception and whether interventions to resolve it (e.g., studying among other students, providing necessary exposure to students from an early grade, and asking for the opinions of majors) will affect motivation toward general medicine. 

The relationship between students’ perception of general medicine as having a broad scope of practice and their motivation toward general medicine needs to be clarified. Whether the broad scope of practice is attractive and motivating or whether motivated people like to diagnose and treat multiple diseases is unknown. Recently in Japan, problem-based learning (PBL) has been conducted as a part of education in general medicine at universities. There have been several study meetings about clinical reasoning by general physicians, and many books have been published on the topic. One study found that general medicine rotations are associated with improved resident ability in clinical reasoning [34]. In contrast, one review showed that PBL was not associated with students’ career choices in a particular specialty in most studies (7 out of 11 studies). However, three studies, excluding a Japanese study, reported a significant increase in the number of graduates who experienced PBL while working in primary care compared to graduates of the non-PBL curriculum [35]. The current evidence suggests that general medicine education by PBL and apprenticeship may improve students’ perception of general medicine and medical residents’ performance [36,37].

In rural contexts, community-based medical education accommodates general medicine education, which can be applied by various professionals. For effective education, general medicine education should include various stakeholders, such as nurses and citizens, to improve the quality of education [30,38]. Rural community-based medical education may lack medical educators because of the lack of physicians in rural contexts. Near-peer learning should be performed to improve rural educational situations. Near-peer learning refers to students instructing other students, evaluating their products or outcomes of learning, and providing assessment and feedback [39]. Near-peer learning could motivate medical students to be self-directed in urban and rural contexts [40,41,42]. The promotion of this learning method could mitigate medical teachers’ fatigue from education. General medicine education in rural contexts could be beneficial for education and clinical practice in rural contexts [43,44]. Therefore, longitudinal studies are needed to determine whether these interventions to develop skills in a broad scope of practice are associated with motivation toward general medicine after its recent establishment in Japan.

This study had some limitations. First, students initially interested in general medicine may have answered the questionnaire, leading to self-selection bias. However, since the response rate was approximately 70%, the results could be considered acceptable. The response rate of the fifth- and sixth-grade students was low. This could be caused by the lack of clinical training because of COVID-19 and the lack of the timing of the explanation of this research face to face. Second, regarding the generalizability of results, it is unclear whether the results can be applied to urban areas since this study was conducted in a rural community in Japan. Third, we used a logic model of human behavior change comprising four stages (knowledge, interest, motivation, and intention); however, further research is needed to determine whether other models may have been more suitable. Fourth, this study’s applicability to other settings must be further investigated. This study was performed in a rural Japanese prefecture; hence, the following study can be performed in other contexts to confirm the difference in the perception of general medicine and family medicine. Finally, since this was a cross-sectional study, causal relationships remain unclear. Further research is required to determine the causal relationships between the relevant variables.

## 5. Conclusions

This study clarified the factors associated with medical students’ preference for general medicine. There was a large gap between the percentage of students interested in and those motivated toward general medicine. This gap was associated with factors such as “Meeting the needs of society”, “Lack of educators”, “Lack of exposure beyond the curriculum”, “Inaccessible”, and “Broad scope of practice”. These findings may provide clues for future interventions in general medical education. General medicine education should accommodate more medical educators in clinical settings and give concrete experience to medical students regarding meeting the needs of society and the broad scope of practice. Further research is needed to investigate the relationship between medical students’ perceptions of general medicine and factors such as general medicine education at universities, their impressions of general medicine, and regional quotas.

## Figures and Tables

**Table 1:** Questionnaire to assess medical students’ perceptions of general medicine.

Theme	Questions
Positive perceptions	Do you think general physicians engage in community-based medicine?
	Do you think general physicians manage preventive care and public health within the local community?
	Do you think general physicians provide home care?
	Do you think general physicians have a broad scope of practice?
	Do you think general physicians have a better balance between clinical practice, education, and research than other organ-based specialists?
	Do you think the need for general physicians is increasing?
	Do you think the diverse aspects of general physicians will lead to future development?
Negative perceptions	Do you think the role of general physicians is specialized or biased toward diagnostics?
	Do you think the role of general physicians is to send patients to appropriate organ-based specialists?
	Do you think general medicine is inaccessible?
	Do you think general medicine is a challenging field?
	Do you think the career path of general physicians is unclear?
	Do you think the field of general medicine is not academic enough?
	Do you think the expertise of general medicine is unclear?
Factors affecting the gap between these perceptions	Do you think there are not enough classes about general medicine in the university curriculum?
	Do you think there is not enough clinical practice for general medicine in the university curriculum?
	Do you think there are few opportunities to be exposed to general medicine outside of the university curriculum?
	Have you ever had a negative perception of general medicine due to other organ-based specialists criticizing general medicine?
	Do you think there is a shortage of educators in general medicine?
	Do you think the quality of educators in general medicine needs to be improved?
	Do you think the policies for general medicine should be improved?
	Did the department of general medicine being new and primary prevented you from being interested in general medicine?
	Do you think there are differences in the education of general medicine and the spread of general medicine among universities and regions?
	Do you think students obligated to work in the prefecture after graduation, such as Chiikiwaku, are forced to be general physicians?

**Table 2:** Characteristics of the participants and their perceptions regarding general medicine, classified by their preferences.

	Knowledge			Interest			Motivation			Intention		
	+ (%)	− (%)	*p*-Value	+ (%)	− (%)	*p*-Value	+ (%)	− (%)	*p*-Value	+ (%)	− (%)	*p*-Value
	*n* = 356	*n* = 134		*n* = 325	*n* = 165		*n* = 181	*n* = 309		*n* = 52	*n* = 438	
**Grade**												
Pre-clinical (Grade 1–4)	240 (67.4)	111 (82.8)	0.001	228 (70.2)	123 (74.5)	0.341	129 (71.3)	222 (71.8)	0.917	31 (59.6)	320 (73.1)	0.051
Clinical (Grade 5,6)	116 (32.6)	23 (17.2)		97 (29.8)	42 (25.5)		52 (28.7)	87 (28.2)		21 (40.4)	118 (26.9)	
**Hometown**												
Shimane	88 (24.9)	21 (15.7)	0.029	78 (24.1)	31 (18.9)	0.207	53 (29.4)	56 (18.2)	0.005	14 (27.5)	95 (21.8)	0.376
Other Prefecture	265 (75.1)	113 (84.3)		245 (75.9)	133 (81.1)		127 (70.6)	251 (81.8)		37 (72.5)	341 (78.2)	
**Sex, male**	176 (49.4)	50 (37.3)	0.019	158 (48.6)	68 (41.2)	0.126	78 (43.1)	148 (47.9)	0.348	22 (42.3)	204 (46.6)	0.659
**Chiikiwaku**	92 (25.8)	13 (9.7)	<0.001	82 (25.2)	23 (13.9)	0.004	54 (29.8)	51 (16.5)	0.001	19 (36.5)	86 (19.6)	0.007

**Table 3:** Students’ perceptions regarding general medicine, classified by their preferences.

	Knowledge			Interest			Motivation			Intention		
	+ (%)	− (%)	*p*-Value	+ (%)	− (%)	*p*-Value	+ (%)	− (%)	*p*-Value	+ (%)	− (%)	*p*-Value
	*n* = 356	*n* = 134		*n* = 325	*n* = 165		*n* = 181	*n* = 309		*n* = 52	*n* = 438	
**Positive perceptions**												
Community-based medicine	329 (92.4)	95 (70.9)	<0.001	303 (93.2)	121 (73.3)	<0.001	170 (93.9)	254 (82.2)	<0.001	49 (94.2)	375 (85.6)	0.129
Preventive care and public health	321 (90.2)	101 (75.4)	<0.001	299 (92.0)	123 (74.5)	<0.001	165 (91.2)	257 (83.2)	0.015	44 (84.6)	378 (86.3)	0.676
Home care	308 (86.5)	85 (63.4)	<0.001	269 (82.8)	124 (75.2)	0.055	152 (84.0)	241 (78.0)	0.127	43 (82.7)	350 (79.9)	0.716
Broad scope of practice	329 (92.4)	103 (76.9)	<0.001	311 (95.7)	121 (73.3)	<0.001	172 (95.0)	260 (84.1)	<0.001	49 (94.2)	383 (87.4)	0.179
Balance between practice, education, and research	182 (51.1)	49 (36.6)	0.004	173 (53.2)	58 (35.2)	<0.001	94 (51.9)	137 (44.3)	0.112	25 (48.1)	206 (47.0)	0.885
Meeting the needs of society	327 (91.9)	107 (79.9)	<0.001	309 (95.1)	125 (75.8)	<0.001	174 (96.1)	260 (84.1)	<0.001	50 (96.2)	384 (87.7)	0.102
Diversity and development	308 (86.5)	96 (71.6)	<0.001	287 (88.3)	117 (70.9)	<0.001	162 (89.5)	242 (78.3)	0.002	48 (92.3)	356 (81.3)	0.053
**Negative perceptions**												
Bias towards diagnostics	146 (41.0)	47 (35.1)	0.254	131 (40.3)	62 (37.6)	0.625	74 (40.9)	119 (38.5)	0.633	24 (46.2)	169 (38.6)	0.297
Sending patients to appropriate speciality	171 (48.0)	60 (44.8)	0.544	160 (49.2)	71 (43.0)	0.214	92 (50.8)	139 (45.0)	0.224	27 (51.9)	204 (46.6)	0.468
Inaccessible	70 (19.7)	27 (20.1)	0.899	59 (18.2)	38 (23.0)	0.23	29 (16.0)	68 (22.0)	0.127	9 (17.3)	88 (20.1)	0.716
Difficulty	277 (77.8)	94 (70.1)	0.098	262 (80.6)	109 (66.1)	0.001	140 (77.3)	231 (74.8)	0.585	35 (67.3)	336 (76.7)	0.17
Ambiguity in career path	212 (59.6)	71 (53.0)	0.218	191 (58.8)	92 (55.8)	0.562	108 (59.7)	175 (56.6)	0.57	38 (73.1)	245 (55.9)	0.018
Not academic	87 (24.4)	25 (18.7)	0.186	70 (21.5)	42 (25.5)	0.363	45 (24.9)	67 (21.7)	0.436	14 (26.9)	98 (22.4)	0.485
Unclear expertise	227 (63.8)	77 (57.5)	0.211	203 (62.5)	101 (61.2)	0.844	111 (61.3)	193 (62.5)	0.847	31 (59.6)	273 (62.3)	0.763
**Factors affecting the gap between these perceptions**												
Lack of classes in the curriculum	190 (53.4)	68 (50.7)	0.613	187 (57.5)	71 (43.0)	0.003	99 (54.7)	159 (51.5)	0.512	31 (59.6)	227 (51.8)	0.307
Lack of clinical practice in the curriculum	159 (44.7)	53 (39.6)	0.357	148 (45.5)	64 (38.8)	0.177	87 (48.1)	125 (40.5)	0.109	31 (59.6)	181 (41.3)	0.017
Lack of exposure beyond the curriculum	154 (43.3)	53 (39.6)	0.474	146 (44.9)	61 (37.0)	0.1	72 (39.8)	135 (43.7)	0.449	21 (40.4)	186 (42.5)	0.882
Criticism from other specialists	91 (25.6)	21 (15.7)	0.022	75 (23.1)	37 (22.4)	0.91	49 (27.1)	63 (20.4)	0.095	21 (40.4)	91 (20.8)	0.003
Lack of educators	211 (59.3)	55 (41.0)	<0.001	192 (59.1)	74 (44.8)	0.003	114 (63.0)	152 (49.2)	0.004	29 (55.8)	237 (54.1)	0.883
Need for improvement in educators’ quality	110 (30.9)	37 (27.6)	0.509	94 (28.9)	53 (32.1)	0.467	59 (32.6)	88 (28.5)	0.359	15 (28.8)	132 (30.1)	1
Immaturity of healthcare policy	135 (37.9)	39 (29.1)	0.073	121 (37.2)	53 (32.1)	0.274	70 (38.7)	104 (33.7)	0.283	24 (46.2)	150 (34.2)	0.094
Immaturity of the field	84 (23.6)	30 (22.4)	0.812	82 (25.2)	32 (19.4)	0.175	50 (27.6)	64 (20.7)	0.096	13 (25.0)	101 (23.1)	0.731
Large regional disparity	247 (69.4)	78 (58.2)	0.024	233 (71.7)	92 (55.8)	0.001	132 (72.9)	193 (62.5)	0.023	39 (75.0)	286 (65.3)	0.214
Relationship with Chiikiwaku	123 (34.6)	36 (26.9)	0.129	100 (30.8)	59 (35.8)	0.307	60 (33.1)	99 (32.0)	0.842	22 (42.3)	137 (31.3)	0.118

**Table 4:** Factors associated with the gap between medical students’ interest in and motivation for general medicine.

Factor	Odds Ratio	95%CI	*p*-Value
**Background of the participants**			
Hometown, Shimane	1.38	0.79–2.40	0.25
Chiikiwaku	1.47	0.83–2.59	0.19
**Questions about medical students’ perceptions regarding general medicine**			
Community-based medicine	1.66	0.69–3.99	0.26
Preventive care and public health	1.09	0.50–2.37	0.83
Home care	0.8	0.45–1.44	0.46
Broad scope of practice	2.36	0.97–5.76	0.059
Balance between practice, education, and research	0.97	0.63–1.49	0.87
Meeting the needs of society	**2.61**	**1.02–6.68**	**0.045**
Diversity and development	1.5	0.78–2.92	0.23
Bias towards diagnostics	0.84	0.54–1.29	0.42
Sending patients to appropriate speciality	1.19	0.79–1.81	0.41
Inaccessible	**0.5**	**0.28–0.88**	**0.016**
Difficulty	0.78	0.47–1.31	0.35
Ambiguity in career path	0.99	0.62–1.58	0.95
Not academic	1.39	0.83–2.32	0.22
Unclear Expertise	0.8	0.50–1.26	0.33
Lack of classes in the curriculum	0.8	0.48–1.34	0.4
Lack of clinical practice in the curriculum	1.58	0.92–2.71	0.098
Lack of exposure beyond the curriculum	**0.62**	**0.39–0.99**	**0.045**
Criticism from other specialists	1.41	0.83–2.40	0.2
Lack of educators	**1.74**	**1.10–2.76**	**0.018**
Needs to improve the quality of educators	1.1	0.65–1.86	0.71
Immaturity of healthcare policy	0.9	0.55–1.48	0.67
Immaturity of the field	1.4	0.84–2.34	0.19
Large regional disparity	1.34	0.82–2.18	0.25
Relationship with Chiikiwaku	0.75	0.47–1.19	0.22

## Data Availability

The datasets used and analyzed during the current study are available from the corresponding author on reasonable request.
